# Bleeding Per Rectum in a Patient with an Amputated Finger

**DOI:** 10.4103/1319-3767.54741

**Published:** 2009-07

**Authors:** Aijaz Hakeem, Hakim Shafi, Shubana Rasool, Muneer Ahmad

**Affiliations:** Departments of Radiodiagnosis, General Surgery, and Gastroenterology, SK Institute of Medical Sciences, Srinagar, Kashmir - 190 011, India

A 42-year-old female who had her right index finger amputated 13 months back now presented to the department of gastroenterology with symptoms of abdominal pain and bleeding per rectum. On examination the patient had iron deficiency anemia and features of cachexia. Upper gastrointestinal (GI) endoscopy and colonoscopy were done but did not reveal any lesion. The patient underwent computed tomography (CT) scan on a Siemens 64-slice CT scanner, which showed multiple rounded, markedly enhancing, small gut (jejunal) lesions, along with an enteroenteric intussusception [Figures 1–3]. A similarly enhancing lesion was also found in the left gluteus medius muscle.

**Figure 1:**
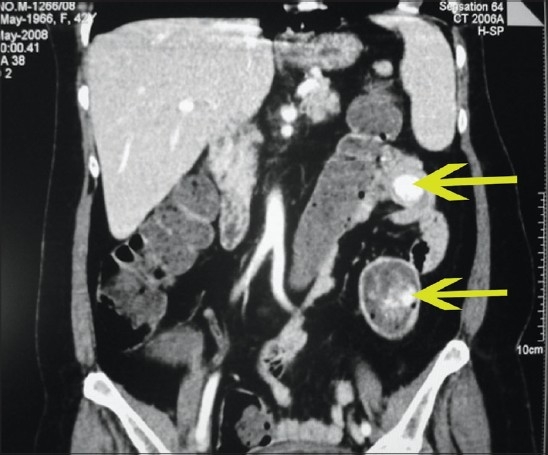
MDCT coronal image showing two markedly enhancing lesions in the jejunum

**Figure 2:**
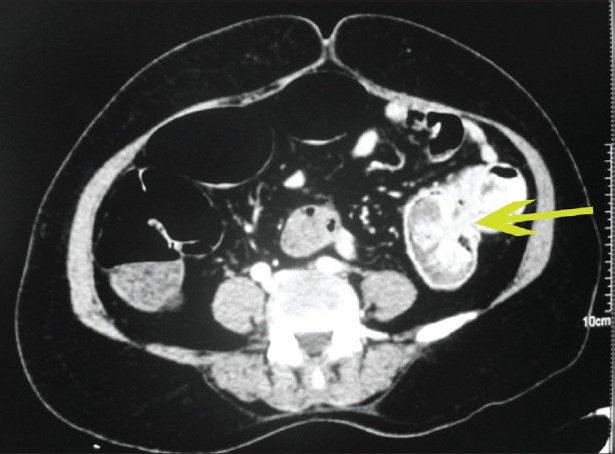
Axial MDCT image showing markedly enhancing lesions in the jejunum

**Figure 3:**
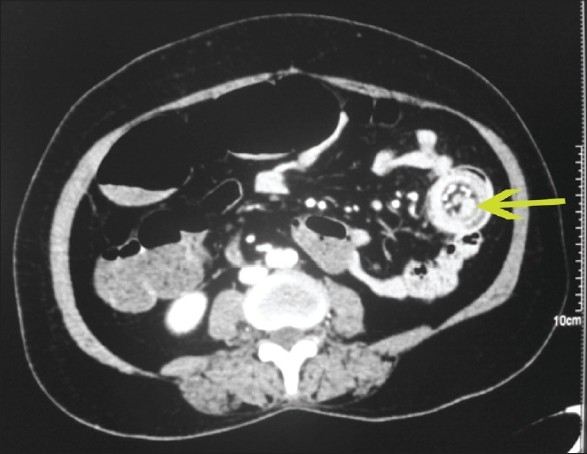
Axial MDCT image showing enteroenteric intussusception, with an enhancing lesion as the lead point

## QUESTIONS

1. What is the probable close differential diagnosis?
2. What is the appropriate diagnostic modality in such patients?
3. What treatment should be offered to this patient?

## ANSWERS

1. Malignant melanoma metastasis to jejunum leading to intussusception.
2. CT enterography.
3. Palliative resection (enterectomy with end-to-end anastomosis), along with chemotherapy and immunotherapy, is the procedure of choice in patients with this condition.

## DISCUSSION

Cutaneous melanoma is a malignancy characterized by a high mortality rate. It can metastasize to all organs, though the GI tract is an unusual metastatic localization. In fact, in 50% of cutaneous melanomas, metastases in the GI tract are diagnosed upon autopsy; in only 5% of cases are they diagnosed clinically.[1,2] The small intestine is the most common site of GI metastases from a cutaneous malignant melanoma. Small bowel metastatic deposits attributed to malignant melanoma are found in 2-5% of patients with malignant melanoma of the skin. Intestinal metastasis from melanoma is usually found to affect the stomach or the colon. Jejunal location is much less frequent[3] and, if present, does not show clinical signs, the diagnosis being made only at autopsy. The patient may present with an intussusception, with a submucosal lesion as the leading point.[4] Intestinal metastases of cutaneous melanomas are linked to a poor prognosis, with an average survival of 6-10 months after surgery.[5,6] Many studies have demonstrated that only surgery can control the chronic anemia related to the bleeding resulting from intestinal melanoma and resolution of the episodes of intestinal subocclusion. Surgery on melanoma metastases, moreover, can guarantee an increase in survival, in addition to an excellent improvement in quality of life.[7–10] At present, palliative resection, along with chemotherapy and immunotherapy, is the procedure of choice in patients with this condition.[11]

Our case had abdominal pain with mild bleeding per rectum, which is extremely rare with metastasis to the intestine.[12,13] We suggest that in any known case of malignant melanoma presenting with abdominal symptoms, with normal endoscopy and colonoscopy, CT scan should be the investigation of choice, especially when 64-slice CT is available. Negative oral contrast should be used as these lesions are markedly enhancing and can blend with positive oral contrast and be missed if the latter is used.
